# Values, trade-offs and ethical choices in climate-related health research

**DOI:** 10.2471/BLT.26.295841

**Published:** 2026-03-01

**Authors:** Jerome Amir Singh

**Affiliations:** a Howard College School of Law, University of KwaZulu-Natal, King George V Avenue, Glenwood, Durban, 4091, South Africa.

Climate change is recognized as one of the greatest threats to human health, with impacts that are measurable and projected to intensify.^1^^–^^3^ Research on mitigation and adaptation has expanded rapidly, alongside growing recognition that climate change is an environmental and economic challenge as well as a threat to population health.^4^ Yet as climate–health research grows, ethical questions about what should be studied, for whom, by whom and through which priority-setting processes, have become increasingly salient. 

Health research is not value-neutral. Decisions about which health outcomes are prioritized, which populations are included, where the benefits of research are expected to accrue, what levels of risk are considered acceptable and how uncertainty is interpreted, inevitably reflect value deliberations.^5^ In the context of climate change, these deliberations carry particular weight. Climate-related health harms are increasingly foreseeable; they are also unequally distributed and in many cases irreversible, creating ethical reasons for priority-setting in research and policy, even where uncertainty remains about timing, magnitude or local impacts.^6^

Climate–health research involves unavoidable trade-offs. Interventions that reduce long-term health risks may impose short-term social or economic costs. Research that documents population-level harms may offer limited direct benefit to the communities studied. Such tensions are not problems that can be resolved simply by producing more evidence or improving data collection. Because climate–health research is conducted under conditions of urgency, uncertainty and inequality, navigating these tensions requires ethical judgement about priorities and trade-offs, not only technical decision-making.^7^

An example of such value-laden trade-offs arises in policy decisions related to energy production and use, which have direct and indirect health consequences. Governments often face choices between sustaining livelihoods and economic stability through continued reliance on established, carbon-intensive energy sources, accepting associated air pollution and climate-related health harms, or prioritizing the protection of lives by accelerating transitions to cleaner energy systems that carry short-term social and economic costs. Evidence can inform the magnitude and distribution of these harms and benefits, but it cannot determine how competing values such as economic and energy security, equity, intergenerational responsibility and population health should be weighed. These values are inherently ethical judgements that shape both policy decisions and the research priorities that inform them.

Values shape how such trade-offs are perceived, weighed and justified. They influence which harms are emphasized, whose interests are prioritized and which uncertainties are considered acceptable. Although values are often treated as implicit background considerations, they influence decisions at every stage of climate–health research. Analyses of global climate and health governance, including across United Nations (UN) system instruments related to climate change, show that certain values recur across policy domains, notably sustainability (often linked to intergenerational justice), equity and justice, and solidarity.^8^ Recent work mapping these values across UN climate, health and humanitarian frameworks highlights how they function as shared reference points for navigating competing interests, time horizons and obligations, rather than as fixed or hierarchical principles. Specifying these values can help clarify why reasonable actors may prioritize differently, and where ethical disagreement requires deliberation rather than technical resolution.

In the practice of health research, additional values are consistently at stake. Commitments to beneficence and non-maleficence require careful consideration of how research may expose participants or communities to harm, particularly where few practical options exist to reduce or remedy those harms. Justice requires attention to fair participant selection, benefit-sharing and priority-setting, especially when research is conducted among populations with constrained resources or adaptive capacity. Respect for people and communities underlines the importance of meaningful engagement, transparency and trust.^9^ Scientific integrity remains essential where research findings are used to inform policy decisions with far-reaching consequences under conditions of uncertainty.^10^

These ethical considerations do not arise in isolation. In some settings, climate–health research is further shaped by constrained funding environments and by contested acceptance of climate science. Such conditions can influence which research questions are pursued, which methods and topics receive support, and which populations remain visible within research agendas. Acknowledging these influences is not a call for political alignment, but an ethical recognition that research priorities are shaped by broader institutional and social contexts, with implications for equity, prevention and global health preparedness.^11^

As climate impacts intensify, the question is no longer whether values shape climate–health research, but whether they are acknowledged and addressed openly. Making values explicit does not resolve all ethical tensions, replace evidence or substitute for legal frameworks. Making values explicit, does provide a shared language for navigating difficult choices in a field that has high stakes for health, equity and future generations.
